# Does social participation reduce the risk of functional disability among older adults in China? A survival analysis using the 2005–2011 waves of the CLHLS data

**DOI:** 10.1186/s12877-018-0903-3

**Published:** 2018-09-21

**Authors:** Min Gao, Zhihong Sa, Yanyu Li, Weijun Zhang, Donghua Tian, Shengfa Zhang, Linni Gu

**Affiliations:** 1 School of Social Development and Public Policy, China Institute of Health, Beijing Normal University, Beijing, 100875 China; 2 School of Sociology, Beijing Normal University, No.19, Xinjiekou wai Street, Beijing, 100875 China; 3 School of Humanities and Social Sciences, North China Electric Power University, Baoding, 071000 China

**Keywords:** Chinese older adults, Onset of functional disability, Social participation

## Abstract

**Background:**

Existing studies in developed countries show that social participation has beneficial effects on the functional ability of older adults, but research on Chinese older people is limited. This study examined the effects of participating in different types of social activities on the onset of functional disability and the underlying behavioral and psychosocial mechanisms among older adults aged 65 and older in China.

**Methods:**

The 2005, 2008, and 2011 waves of the Chinese Longitudinal Health Longevity Study were used. Life table analysis and discrete time hazard models were adopted to examine the relationship between social participation and functional disability. Social participation was defined as the frequencies of engaging in group leisure-time activities (i.e., playing cards/mahjong) and organized social activities, involving in informal social interactions (i.e., number of siblings frequently visited), and participating in paid jobs. Extensive social participation was measured by a composite index by adding up the four types of social activities that an older person was engaged in.

**Results:**

After controlling for the effect of socio-demographic characteristics, health status, and health behavioral factors, extensive social participation is associated with a significant reduced risk for the onset of functional disability (hazard ratio [HR] = 0.92, *p* < 0.001). Different types of social participation affect the risk of functional decline through different mechanisms. Frequent playing of cards/mahjong is a protective factor for functional decline (HR = 0.78, *p* < 0.001), and the relationship is partially mediated by cognitive ability and positive emotions (accounting for 18.9% and 7.0% of the association, respectively). Frequent participation in organized social activities is significantly related to a reduced risk of functional decline (HR = 0.78, *p* < 0.001), and the association is mediated by physical exercises and cognitive ability (accounting for 25.7% and 17.7% of the association, respectively). Frequent visits from siblings has a strong inverse relationship with functional decline (HR = 0.75, *p* < 0.001). However, no significant association between paid job and functional decline is observed.

**Conclusion:**

Extensive social participation, regular engagement in group leisure-time activities, organized social activities, and informal social interactions in particular may have beneficial effects on the functional health of older adults through behavioral and psychosocial pathways. The findings shed light for the importance of promoting social participation among older adults.

## Background

China has entered a period of accelerated population aging that is accompanied by an increasing prevalence of chronic diseases and functional disability among older adults. In 2017, people aged 60 and over accounted for 17.3% of China’s total population of 1.39 billion [1]. In 2013, more than 100 million older adults had at least one chronic noncommunicable disease, of whom more than 37 million had significant declines in physical function [2, 3]. The number of older adults with functional disability is expected to increase to 66 million by 2050 [4]. The large size of older population with chronic diseases and functional disability has created a tremendous challenge for China’s health and social care systems. Encouraging social participation among older adults is an essential part of the active aging strategy [5]. In 2002, the United Nations considered “active aging” a policy framework for addressing population aging in the 21st century. The Chinese government has also adopted the policy of active aging as one of the important means of achieving successful aging for older people.

Numerous studies in developed countries show that social participation has beneficial effects on various health outcomes, including mortality, morbidities, psychological well-being, functional and cognitive abilities, and quality of life [6–8]. Recent studies have also indicated that the relationship between social participation and functional disability varies by participating in different types of social activities or organizations [8]. However, the underlying mechanisms through which different types of social participation affect the risk of functional disability among older adults are not well understood [9].

Despite the potential importance of social participation for the various health outcomes of older adults, existing studies among Chinese older adults tend to focus on the relationship between social participation and mental health or mortality [10, 11]. Only a few studies focus on the relationship between social participation and functional disability. The studies that used cross-sectional data reveal that participation in social activities is inversely associated with functional disability among Chinese older adults [12, 13]. However, the findings based on cross-sectional data may suffer from reverse causality. Moreover, the social and structural contexts of social participation among older adults in China are different from those in developed countries. The Chinese cultural tradition of familism encourages bonds within family and kinship, and civil society in China is still in its early stage of development. Thus, chances for social participation for Chinese older people are rather limited. The present study aimed to investigate the effects of participation in various types of social activities on functional decline and the underlying behavioral and psychosocial mechanisms among Chinese adults aged 65 years and older. This study improved upon previous studies in China by using large representative longitudinal data collected between 2005 and 2011, examining social activities and social relationships, and employing a survival analysis method that treats social participation and the mediating factors as time-varying variables.

### Social participation and functional disability

The influence of social participation on functional ability has been widely discussed in developed countries. According to social integration theory, social engagement gives meaning to an individual’s life by enabling him or her to participate in it fully, to be obligated, to feel attached to one’s community, and to feel fulfilled, which are all beneficial to health. Social relationship can also shape health-related resources that are available and increase motivation and social pressure to take good care of one’s health [14, 15].

In support of social integration theory, existing studies in developed countries consistently show that extensive engagement in social activities is associated with a low likelihood of functional disability among older adults [16–19]. For instance, a study on older Japanese reveals that membership in multiple social organizations is associated with a reduced risk of having incident functional disability possibly because participation in various social activities enables older people to take on multiple social roles [17]. Moreover, these studies indicate that social participation benefits the functional ability of older adults through psychosocial, behavioral, and physiological pathways. First, engagements in social activities may benefit the health of older adults by helping them find social and spiritual supports, thereby reducing mental health problems. Studies provide sufficient evidence that high levels of social participation reduce the likelihood of having mental health problems [20] and self-destructive habits [21], and that mental distress has negative effects on physical health. Moreover, involvement in social activities can help older adults lower the risk of functional disability by maintaining cognitive ability [15]. Second, the underlying linkage between social participation and one’s functional health can be explained by engaging in protective health behaviors [22]. For instance, participation in community voluntary works can benefit the functional health of older adults by staying physically active [23]. Third, social participation may exert direct physiological benefits, such as buffering stress, boosting host resistance, and lowering the biomarkers of disease risks [24].

While most studies use an aggregated indicator of social participation (i.e., number of social activities) [25] or focus on one of its particular types, recent studies have also shown that the relationship between social participation and functional decline differs by types of social activities probably because different social activities play different roles in health prevention [17]. Engagement in leisure-time activities may encourage people to stay physically active and reduce the risk of developing functional disabilities [26]. In addition, participating in organized social activities, such as church attendance and group voluntary activities, could confer health-enhancing benefits through psychosocial pathways, such as having considerable chances to socialize with others aside from family members and obtaining a sense of motivation, inner direction, and purposefulness [27]. A longitudinal study indicates that older adults who engage in volunteering activities have good self-rated health, few depressive symptoms, and good functional abilities possibly because volunteering provides them a chance to take on new roles that are psychologically rewarding [28]. Furthermore, participating in productive activities, such as paid work, is negatively related to the onset of functional decline probably by preserving one's physical and cognitive abilities [29]. Engagement in paid work also means having considerable individual/social resources, which may help in maintaining good health [30]. Although the empirical investigations into the mechanisms through which different types of social participation affect the risk of functional disability among older adults can provide evidence for health promotion, this important issue is understudied in the existing literature.

### Social participation and functional disability in China

Research on the relationship between social participation and functional disability among Chinese older adults is limited. A cross-sectional study based on the nationally representative data reveals that the frequency of participation in various social activities, such as interacting with friends, playing mahjong, volunteering, and other activities, is positively related to the functional ability of older adults. Positive social interactions gained from involving in these activities may provide one of the explanations for the beneficial effects of social participation on functional ability [13]. Another cross-sectional study contends that re-entering into the labor force after retirement is positively related to the functional health of Chinese older adults [31]. These studies provide important evidence for the relationship between social participation and functional health among Chinese older adults, but research based on cross-sectional data may have the problem of reverse causality. Losses in physical function and self-care capacities may lead to reduced social participation; hence, social participation may be more of a consequence of functional disability than it is a cause.

The social and structural contexts of social participation among older adults in China are different from those in developed countries, and the observed relationship between various types of social participation and functional disability in developed countries may not be applicable to Chinese older adults. The Chinese cultural tradition of familism encourages bonds within family and kinship. Thus, informal social interactions among immediate family members and relatives are the most important aspects of social relationships for Chinese older adults. Civil society is still at its early stage of development such that voluntary activities organized by social organizations or self-help groups are rather limited. Cultural and leisure-time activities, such as group dancing, singing, and playing cards/mahjong that are organized by older adults themselves, and formal social or voluntary activities, such as health lectures, trainings on using smartphones, and security patrols organized by residential committees, are the major forms of social activities that Chinese older adults are involved in [32]. Although participation in paid work is an indispensable part of social participation among Chinese older adults, their level of participation in the paid employment is low. China has an early mandatory retirement age such that men retire at 60 years old and women retire between 50 and 55 years old. The opportunities for relocating paid jobs are rather limited among retirees. Although some studies show that participation in paid job is beneficial to health among older adults in China, other studies indicate that Chinese older adults who remain in the paid job after retirement tend to have insufficient financial support and poor health conditions [33, 34]. Older adults who participate in paid jobs available to them after retirement are likely to experience burnout that offsets the beneficial effects of employment on health. On the basis of the existing literature and Chinese social and cultural backgrounds, the present study hypothesized that informal social interactions, group leisure-time activities, and organized social activities are negatively related to functional decline and that paid work is not related to functional ability among Chinese older adults. We also anticipated that extensive social participation has a strong negative relationship with functional decline because taking on multiple social roles may have overlapping health benefits.

## Methods

### Data

Data for this study were obtained from the 2005, 2008, and 2011 waves of the Chinese Longitudinal Healthy Longevity Survey (CLHLS). The CLHLS is a randomly selected sample of rural and urban older adults in 631 countries/cities of 22 provinces, which represents 85% of the total population in China. A detailed description of the sampling design and data quality of the CLHLS has been reported elsewhere [35]. The 1998 baseline and 2000 follow-up surveys of the CLHLS included approximately 10,000 respondents aged 80 years and over. The CLHLS started to interview younger-old persons in 2002. At each wave, survivors were re-interviewed, and the deceased interviewees were replaced by additional participants. The CLHLS collected information on socio-demographic characteristics, physical and mental health status, chronic diseases, family and social supports, and health behaviors.

This study used the fourth, fifth, and sixth waves because the wording of the questions on social participation and functional ability was consistent in the three waves. The CLHLS conducted face-to-face interviews with 15,638, 16,540, and 9765 individuals in 2005 (baseline), 2008 (Time 2), and 2011 (Time 3), respectively. To select an initially nondisabled group, we excluded 25 respondents whose age was below 65 and 3929 respondents who reported having problems with at least one activity of daily living in 2005. We also dropped 840 cases with missing information for independent and dependent variables, leaving a baseline sample of 10,844. After merging the baseline data with 2008 and 2011 waves, the number of respondents who survived and were surveyed in all three waves was 3731 (38.6% of the baseline sample). Compared with those who dropped out of the sample, the remaining participants were significantly more likely to be younger and married, reside in rural areas, have more children, have better education and cognitive and emotional conditions, and engage in all types of social activities and informal social interactions more actively (results shown in Appendix). Therefore, the observed association between social participation and functional disability in the present study may be underestimated.

### Variables

#### Functional disability

Functional disability was measured by six activities (i.e., eating, dressing, indoor mobility, bathing, using the toilet, and continence) from the activities of daily living (ADL) scale. Individuals were asked if she/he had any difficulties with each of the activities. This study focused on the onset of functional disability. Respondents who reported having had no ADL problem at the baseline but experienced some difficulties with or were unable to perform at least one of the ADL activities at Time 2 or Time 3 were defined to have functional disability (coded as 1), and those who reported having had no ADL problem between 2005 and 2011 were defined to have no functional disability (coded as 0). This definition of ADL disability was consistent with that in previous studies [36, 37].

The survey further asked functional disabled respondents about the duration of being unable to perform the activities of daily living at each wave. On the basis of the information, we calculated age at which a respondent became functionally disabled. An individual was considered to have an onset of ADL disability if she/he changed from the status of having no ADL problems to having at least one ADL problem at a certain age.

#### Social participation

Given the social context for the social participation of Chinese older adults and the data limitation, we measured social participation with four indicators, including frequencies of engagement in group leisure-time activities (i.e., playing cards/mahjong) and organized social activities, informal social interactions (i.e., number of siblings frequently visited), and participation in paid jobs. The participants were asked about how often they played cards/mahjong and engaged in organized social activities. Each of the question has five responses: almost every day (coded as 4), not every day but at least once in a week (coded as 3), not every week but once in a month (coded as 2), not every month but sometimes (coded as 1), and never (coded as 0). Informal social interactions were measured by a continuous variable reflecting the number of living siblings frequently visited. Participation in paid work was measured by a dichotomous variable reflecting one’s involvement in a paid job (coded as 1) or not (coded as 0). To further assess the influence of extensive participation in social activities on functional health, we used a composite measure of social participation by adding up the four types of social activities that an older person was engaged in. Each activity was coded as a dichotomous variable reflecting whether an individual participated or not.

#### Mediating variables

To assess the behavioral and psychosocial pathways through which social participation affected the risk of functional decline, health behaviors and cognitive and emotional health conditions were considered potential mediating factors. Physical exercise was used as an indicator of health behavior and was dichotomized into “not doing exercise” (coded as 0) and “doing exercise” (coded as 1). Cognitive ability and positive emotions were included as indicators of psychosocial factors. We measured cognitive ability following the Chinese version of the Mini-Mental Status Examination Index. The range for the cognitive ability index (Cronbach’s *α* = 0.86) was between 0 and 23, with high scores reflecting good cognitive ability. Positive emotions were assessed by three questions: “Do you usually feel nervous or afraid?,” “Do you usually feel lonely?,” and “Do you usually feel more and more useless?” The response for each of the questions ranged between “never” (coded as 5) and “always” (coded as 1). We created a composite measure by summing up the three variables (ranging between 3 and 15), with high scores indicating good emotional condition.

#### Covariates

Three sets of covariates related to functional ability were included in the analysis. The first set of covariates comprised the indicators of demographic characteristics such as age, gender (male coded as 1), marital status (being married coded as 1), and rural/urban residence (urban coded as 1). Age was classified into six categories: 65–69, 70–74, 75–79, 80–84, 85–89, and 90 and above.

The second set of covariates included socioeconomic status, measured by education and self-perceived economic conditions. Given the relatively low level of education among Chinese older adults, education was classified into two categories: illiterate (coded as 0) and literate (coded as 1). The self-perceived economic condition was assessed by the question “How do you rate your economic status compared with other local people?” The variable included three categories: poor, fair, and good.

The third set of covariates consisted of self-rated health status and informal social support. The respondents were asked if they were ever diagnosed with any of the 15 chronic conditions, namely, hypertension, diabetes, heart diseases, stroke (or cardiovascular disease), bronchitis, tuberculosis, cataract, glaucoma, cancer, prostate tumor, gastric or duodenal ulcer, Parkinson’s disease, bedsore, arthritis, and dementia. The number of chronic diseases was calculated by summing up the diseases they had been diagnosed with. Informal social support was indicated by a continuous variable reflecting the number of living children.

### Statistical analysis

The survival analysis method was used given its advantage in predicting the influence of social participation on the onset of functional disability. The 7-year follow-up period was divided into 3-month intervals, and the respondents who did not have incident functional disability during the period were censored. First, a description of sample characteristics was presented. Second, we estimated the bivariate association between the different types of social participation and the onset of functional disability using life table analyses. Third, given that the dependent variable (i.e., time for the onset of functional disability) was a dichotomous measure, we used discrete hazard models to estimate the effect of social participation on the onset of functional disability after adjusting for the mediating factors and other covariates. A nested modeling approach was used to assess the mediating effect of the behavioral and psychosocial factors in the relationship between social participation and functional decline. We sequentially added behavioral and psychosocial factors in models 3 and 4 to identify the changes in HR estimates associated with various types of social participation. Finally, we calculated the mediating effects by using Sobel’s test. The proportion of risk reduction for the onset of functional disability that was explained by each potential mediating factor was computed as follows: [(HR_basic model_ − HR_adjusted model_) / (HR_basic model_ − 1)] * 100% [38]. Given the possible reciprocal relationship between social participation and functional disability, social participation, behavioral and psychosocial mediating factors, and some covariates were treated as time-varying variables, which can provide accurate prediction for the relationship between social participation and functional decline.

## Results

### Sample characteristics

Table 1 presents the distribution of sample characteristics. Respondents aged 80 years or older accounted for 44.6% of the entire sample. More than half of the respondents were women (51.7%), unmarried (51.2%), and residents of rural areas (53.2%). A total of 44.2% of the older adults were illiterate. Most of the respondents perceived their economic conditions as “fair” (68%), whereas 16% considered their economic status as “poor.” The average number of living children was 3.9. The respondents reported having 1.2 diagnosed chronic diseases on average.

**Table 1:** Description of sample characteristics among older adults aged 65 and over, CLHLS 2005, 2008, and 2011

Variables	% or mean (*s.d.*)
Social participation	
Extensive social participation (0–4)	0.9 (0.9)
Playing cards/mahjong (0–4)	0.6 (1.3)
Organized social activities (0–4)	0.4 (0.9)
Number of siblings frequently visited (0–8)	0.7 (1.3)
Paid job	
Not involved	94.2
Involved	5.8
Mediating variables	
Health behavior	
Physical exercise	
No	57.2
Yes	42.8
Psychosocial factors	
Cognitive ability (0–23)	20.0 (3.9)
Positive emotions (3–15)	11.5 (2.4)
Control variables	
Rural/urban residence	
Rural	53.2
Urban	46.8
Gender	
Female	51.7
Male	48.3
Age	
65–69	9.0
70–74	24.0
75–79	22.3
80–84	16.3
85–89	12.0
90 and above	16.3
Marital status	
Widowed/divorced/never married	51.2
Married	48.8
Education	
Illiterate	44.2
Literate	55.8
Self-perceived economic conditions	
Poor	16.0
Fair	68.0
Good	16.0
Number of living children	3.9 (1.8)
Number of chronic diseases	1.2 (1.3)
N	104,468

The overall level of social participation was low with an average number of social activities participated of 0.9 out of a total score of 4 (Table 1). The mean scores for group leisure-time activities (i.e., playing cards/mahjong) and organized social activities were 0.6 and 0.4 (out of a range between 0 and 4), respectively. The level of informal social interactions was also low with an average number of siblings frequently visited of 0.7 (out of a range of 0 and 8). Only 5.8% of the respondents were involved in paid jobs. Table 1 also shows that 42.8% of the respondents engaged in physical exercise. The average scores of the cognitive ability and positive emotion indices were 20 (for a range between 0 and 23) and 11.5 (for a range between 3 and 15), respectively, reflecting good overall cognitive and emotional conditions among older adults.

### Life table analyses

**Fig. 1:**
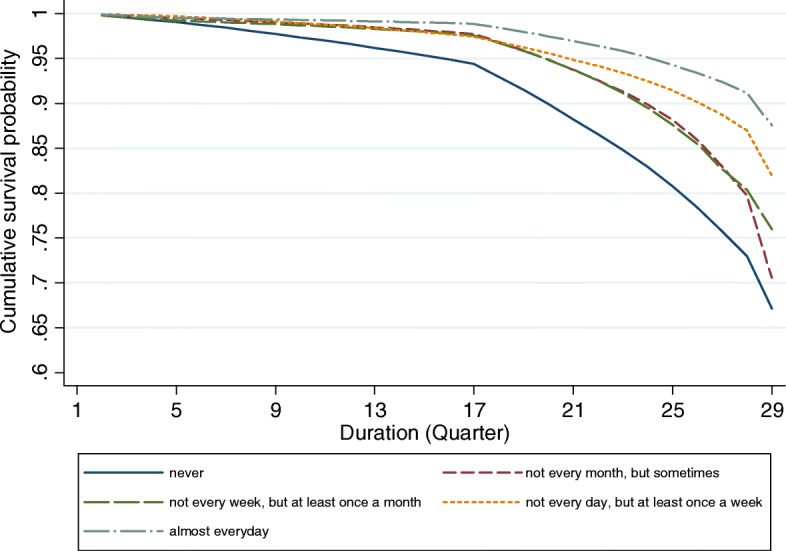
Cumulative survival for the onset of functional disability by participation in group leisure-time activities

**Fig. 2:**
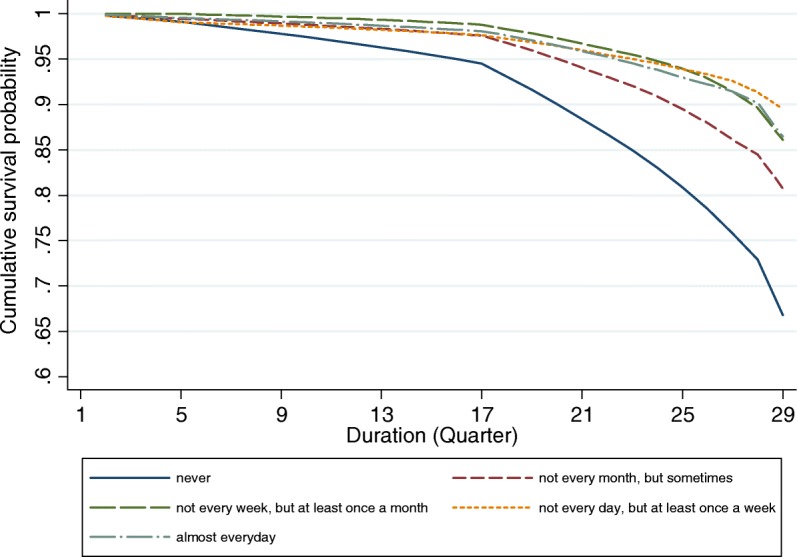
Cumulative survival for the onset of functional disability by participation in organized social activities

**Fig. 3:**
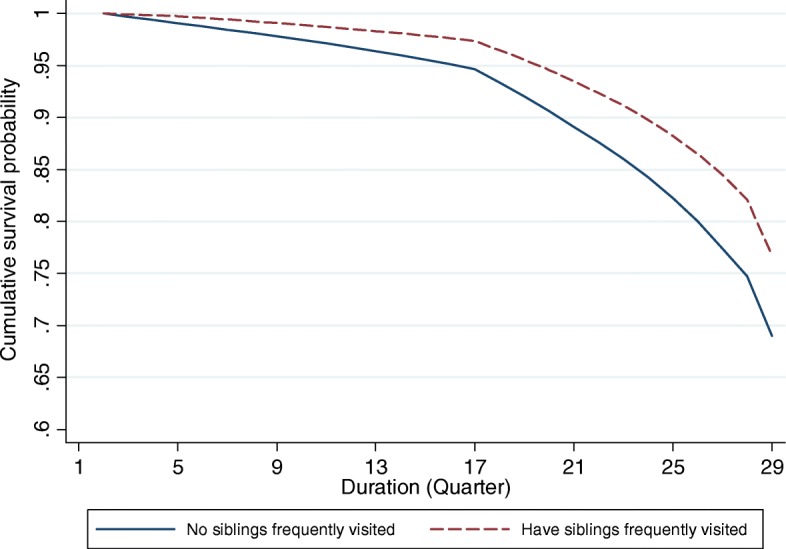
Cumulative survival for the onset of functional disability by informal social interactions

**Fig. 4:**
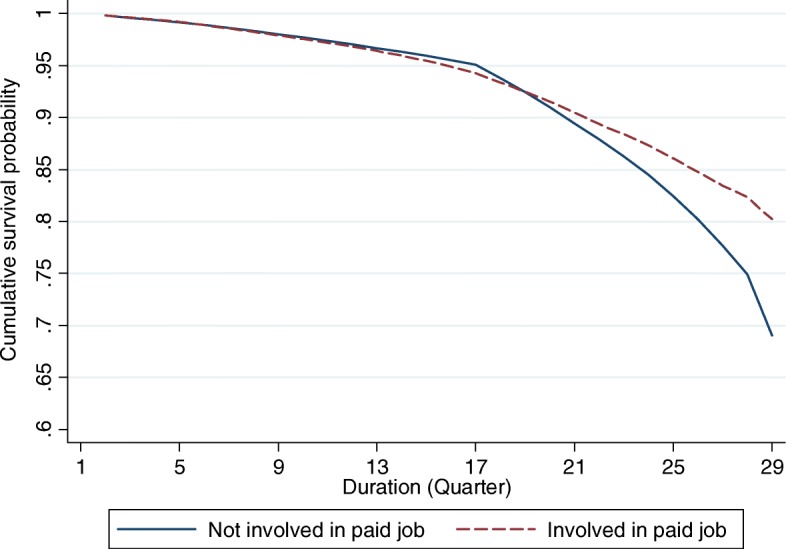
Cumulative survival for the onset of functional disability by participation in paid job

Figures 1, 2, 3 and 4 provide graphical displays of cumulative survival probability for the onset of functional disability by different types of social participation among older adults over the 7-year period. The figures illustrate that the cumulative survival steeply declined among those who lack of or had low levels of social participation, and that the pattern was consistent for four types of social activities. Particularly, Figs. 1 and 2 depict that the cumulative survival for older persons who never played cards/mahjong or who did not participate in organized social activities declined more rapidly than that for those who participated in these activities frequently. Figure 3 shows that people who were not frequently visited by siblings had a higher probability of having functional disability compared with those whose siblings frequently visited. Figure 4 indicates that the cumulative survival probability for older persons who were not involved in paid jobs was smaller than that for people who were involved in paid jobs.

### Regression results

The results from the univariate discrete time hazard regression models were consistent with the results from life table analyses (Table 2)**.** The first column of Table 2 shows that extensive social participation had a strong negative association with the onset of functional disability. Moreover, playing cards/mahjong, engaging in organized social activities, and being frequently visited by siblings were all significantly related to the reduced risk of having incident functional disability, whereas participation in paid jobs was not significantly associated with the risk of functional decline.

**Table 2:** Discrete time hazard models predicting the onset of functional disability

	Crude	Model 1	Model 2	Model 3	Model 4
	HRs	HRs	HRs	HRs	HRs
Extensive social participation	0.62^***^	0.92^***^	–	–	–
Playing cards/mahjong	0.71^***^	–	0.78^***^	0.79^***^	0.84^***^
Organized social activities	0.72^***^	–	0.78^***^	0.83^**^	0.86^*^
Paid job (vs. Not involved)	0.86	–	0.92	0.97	1.13
Number of siblings frequently visited	0.59^***^	–	0.75^***^	0.75^***^	0.76^***^
Residence (vs. Rural)	1.65^***^	1.78^***^	1.61^***^	1.74^***^	1.78^***^
Gender (vs. Female)	0.84	1.06	0.93	0.95	1.07
Age (vs. 65–69)					
70–74	4.39^**^	4.11^**^	3.88^**^	3.95^**^	3.70^*^
75–79	6.13^***^	5.27^**^	4.95^**^	5.04^**^	4.44^**^
80–84	11.58^***^	9.41^***^	9.19^***^	9.22^***^	7.80^***^
85–89	12.58^***^	8.89^***^	10.09^***^	9.72^***^	7.16^***^
90 and above	27.23^***^	17.12^***^	21.26^***^	20.09^***^	13.63^***^
Educated (vs. Illiteracy)	0.80^*^	1.33^**^	1.15	1.23^*^	1.37^**^
Married (vs. Unmarried)	0.58^***^	1.20	1.12	1.12	1.22
Self-perceived economic conditions (vs. Poor)					
Fair	0.77^*^	1.04	0.82	0.83	1.07
Good	0.82	1.13	0.76	0.78	1.16
Number of living children	1.01	1.02	1.03	1.02	1.03
Number of chronic diseases	1.24^***^	1.26^***^	1.24^***^	1.24^***^	1.25^***^
Physical exercise (vs. No)	0.47^***^	0.59^***^		0.51^***^	0.61^***^
Cognitive ability index	0.88^***^	0.92^***^			0.92^***^
Positive emotion index	0.85^***^	0.89^***^			0.89^***^
Constant		0.01^***^	0.00^***^	0.00^***^	0.00^***^
N	104,468	104,468	104,468	104,468	104,468

^*^p < 0.05; ^**^p < 0.01; ^***^p < 0.001

In multivariate discrete time hazard regression analyses (Table 2), extensive social participation was significantly associated with a decreased risk of functional decline after adjusting for socio-demographic characteristics, health status, and behavioral and psychosocial factors (HR = 0.92, *p* < 0.001) (**Model 1**). In **Model 2**, regular participation in cards/mahjong playing (HR = 0.78, *p* < 0.001) and organized social activities (HR = 0.78, *p* < 0.001) and frequent visits by several siblings (HR =0 .75, *p* < 0.001) were all significantly associated with decreased risks of functional decline after controlling for socio-demographic characteristics and health status. However, involving in paid jobs was not significantly related to functional decline. **Model 3** indicates that, after adding physical exercise in the model, the significant association between participation in group leisure-time activities (HR = 0.79, *p* < 0.001) and organized social activities (HR = 0.83, *p* < 0.01) with functional disability still remained, but the magnitude of the associations was slightly reduced. **Model 4** shows that, when cognitive ability and positive emotions were added in the model, the magnitude of the association between participation in group leisure-time activities (HR = 0.84, *p* < 0.001) and organized social activities (HR = 0.86, *p* < 0.05) with functional decline was further reduced but still remained significant. A significant inverse relationship between frequent visits by siblings (HR = 0.76, *p* < 0.001) and the onset of functional disability was observed even after controlling for the effects of behavioral and psychosocial factors and other covariates, and the magnitude of the association remained similar across **Models 2** and **4**.

Moreover, engagement in physical exercise (HR = 0.61, *p* < 0.001) was associated with a low risk of functional decline. Having good cognitive ability (HR = 0.92, *p* < 0.001) and a high degree of positive emotions (HR = 0.89, *p* < 0.001) were both significantly related to a reduced risk of functional disability (**Model 4**). **Model 4** also indicates that older adults who were 70 years or older or literate (HR = 1.37, *p* < 0.01), who lived in urban areas (HR = 1.78, *p* < 0.001), or who had more chronic diseases (HR = 1.25, *p* < 0.001) tended to have higher risks for the onset of functional disability than those in reference groups.

### Mediating effect analyses

We further explored the potential mediating effects of behavioral and psychosocial factors on the relationship between various types of social activities and functional decline. Table 3 reveals that cognitive ability had the strongest mediating effect for the association between playing cards/mahjong and the onset of functional disability, accounting for 18.9% of the association, followed by positive emotions (7.0%) and physical exercise (5.4%). The inverse relationship between engaging in organized social activities and the risk of functional decline was largely explained by physical exercise (25.7%) and cognitive ability (17.7%). Behavioral and psychosocial factors also had significant mediating effects for the association between the number of siblings frequently visited and the onset of functional disability, but the magnitude of the mediating effect was modest. Thus, further studies are needed to explore possible intervening factors between informal social interactions and functional disability among older adults.

**Table 3:** Mediating effects between social participation and the onset of functional disability

	Crude HRs	Physical exercise		Cognitive ability		Positive emotions	
		Adjusted HRs	Mediating effect (%)	Adjusted HRs	Mediating effect (%)	Adjusted HRs	Mediating effect (%)
Playing cards/mahjong	0.78^***^	0.79^***^	5.4	0.82^***^	18.9	0.79^***^	7.0
Organized social activity	0.78^***^	0.83^***^	25.7	0.82^***^	17.7	0.78^***^	0.3
Number of siblings frequently visited	0.75^***^	0.75^***^	1.9	0.76^***^	2.8	0.76^***^	1.4
Paid job^a^	0.91	0.96	–	1.03	–	0.97	–

^***^p < 0.001

^a^The variable paid job was not significantly associated with functional disability, so the mediating effect was not calculated

## Discussion

Although social participation is related to low risks of having functional disability among older adults, the mechanisms underlying this inverse association are unclear. To the best of our knowledge, this study is one of the few studies that explores the relationship between various types of social activities and the onset of functional disability and the underlying behavioral and psychosocial mechanisms among older adults in China. In support of our hypothesis, the present study demonstrates that extensive social participation had a strong negative relationship with the risk of functional decline. The beneficial effects of participating in various social activities on functional ability are consistent with those in existing studies in developed countries [17, 18]. High levels of social participation are associated with low risks of functional decline by enabling older people to take on multiple social roles that are psychologically rewarding [12]. Moreover, social participation may exert direct health benefits by keeping them physically [23] or cognitively active [39].

The present study also reveals that frequent participation in group leisure-time activities, such as playing cards/mahjong, is protective against functional decline among older adults. The finding is consistent with a previous longitudinal study of Chinese older adults, which also indicates that involving in entertaining activities is associated with a low likelihood of having incidence of disability and death [40]. In this study, the association between playing cards/mahjong and the onset of functional disability is partially mediated by cognitive ability (accounting for 18.9% of the association). Playing cards/mahjong is an important part of leisure-time activities among Chinese older adults. The cognitively demanding nature of these activities might help them exercise reaction ability and memory, thereby delaying deterioration. Existing research provides evidence that the higher the level of participating in intellectual activities, the greater the benefit for cognitive ability among older people [41]. Studies also show that playing mahjong or cards can produce consistent gains across many cognitive performance measures among older adults [42]. Improvements in cognitive ability may result in changes in behaviors that promote broad-based engagement in functional activities [43]. Our further analysis reveals that older adults who play cards/mahjong more frequently are also significantly more likely to be involved in physical exercise compared with those who do not play cards/mahjong or play less frequently.

In addition, engagement in group leisure-time activities may be helpful for older adults in resolving negative emotions through positive social interactions. This study indicates that positive emotions explain 7% of the association between playing cards/mahjong and the onset of functional disability. A previous study reveals that engagement in group leisure-time activities is an effective way to alleviate negative emotions and obtain psychological well-being [44]. A Chinese study also shows that playing chess or cards is protective against the negative effects of stressful life events [45]. People may be willing to talk about problems they encounter with and try to seek emotional supports while playing with friends or relatives. Engagement in leisure-time activities is also associated with a low level of depression [46], which is a known risk factor for functional disability among older adults.

The present study also demonstrates that engagement in organized social activities is protective against the risk for the onset of functional disability among older people in China mainly by remaining physically active and maintaining cognitive ability. Some studies show that participation in organized social activities can encourage older people to do sufficient physical exercises together with others or on their own [13, 29]. Moreover, social networks built by engaging in organized social activities increase the motivation of older adults to take good care of one’s health, provide considerable opportunities for companionship, and give meaning to life, which are all beneficial to physical and emotional health. Participating in group dancing and singing, listening to health promotion lectures, or playing cards/mahjong and attending workshops on how to use computers and cell phones are the most popular social activities that Chinese older people engage in. The former activities may help older adults gain significant health-related information and self-efficacy for keeping physically or cognitively active, whereas the latter activities may stimulate the brain functioning of older adults in the process of playing, learning, and memorizing new information and practicing new skills [41].

Despite of the beneficial effects of engaging in organized social activities on functional ability, participation in organized social activities is uncommon among older people in China. In our study, only 17.6% of the respondents report having engaged in organized social activities. Civil society is still in its early stage of development in China, and cultural, recreational, and voluntary activities organized by residential committees are the major forms of organized social activities engaging older people. Studies have indicated that most Chinese older adults are willing to participate in community life, but barriers, such as social exclusion, age discrimination, limited social connectedness, lack of access to information, and low socioeconomic status, hinder their participation. In addition, some of the formally organized social activities aim to accomplish various governmental goals, which may not be able to increase motivation and encourage active participation [47]. Thus far, the elite group of older adults tends to be the most socially active ones, and those with low socioeconomic status generally have limited participation in organized social activities.

Being frequently visited by siblings is significantly associated with reduced risks for the onset of functional disability. As an indispensable part of the social life among Chinese older adults, frequent visits from siblings appear to provide them with emotional support [48]. Accordingly, the risk of functional disability among older adults may be further reduced. The mediating effect of behavioral and psychosocial factors for the association between informal social interactions and functional disability is significant but weak. Thus, informal social interactions may affect the risk of functional disability among older adults through other mechanisms, such as by reducing stress.

Unlike some existing studies in China and other countries, which contend that engagement in paid work in old age benefits functional health [30, 49], our study shows that paid job is not significantly related to the functional disability of older adults. In this study, only 5.3% of the respondents have engaged in paid jobs during the 7-year period. One possible reason for this lack of association is that, in a social context with early mandatory retirement age and limited access to well-positioned paid jobs, the participation of older adults in paid jobs after retirement is likely to be out of financial necessity [47]. Moreover, although the engagement of older adults in paid jobs could bring them beneficial psychological effects [31], it may also exert adverse health consequences that offset the gains from social participation.

The present study has some strengths, such as the use of large panel data, which allows for the investigation of the relationship between change in various types of social participation and the onset of functional disability and its underlying mechanisms. Nonetheless, our study has certain limitations. First, the findings cannot be generalized to the entire old population in China because the sample only represents 85% of the total population. Second, although we consider the reciprocal relationship between social participation and functional ability by treating social participation and medicating factors as time-varying variables, possible reverse causality cannot be fully avoided. Third, measures of social activities are somewhat limited such that they cannot capture the variety of social activities that Chinese older people engage in. Fourth, future research should include additional intervening variables, such as mastery over life and stress, which may mediate the relationship between social participation and the onset of functional disability. Finally, the relatively high attrition rate of the panel data may bias the observed relationship between social participation and functional disability. Older adults who remain in the sample are significantly more likely to participate in social activities than those who are excluded in the follow-up surveys. Thus, the observed relationship between social participation and functional disability may be underestimated.

## Conclusion

In conclusion, extensive social participation, regular engagement in group leisure-time activities and organized social activities, and active involvement in informal social interactions may provide functional health benefits to Chinese older adults through behavioral and psychosocial pathways to a certain extent. Clarifying the effect of different types of social participation on the onset of functional disability may shed important light on the development of policies and intervention programs to promote active social participation among older adults. Given the limited access of Chinese older adults in participating in social life, considerable efforts are needed by the government to develop supportive and effective policies to eliminate ageism and structural barriers for the broad social participation of older adults. Community and social organizations should play highly active roles in building a lively community life for older people.

## Appendix

**Table 4:** Baseline characteristics of survivors and respondents who dropped out between 2005 and 2011

	Survivors in 2011 (*N* = 3731)	Drop-outs between 2005 and 2011 (*N* = 7113)	*p*-value^1^
	% or mean (*s.d.*)	% or mean (*s.d.*)	
Playing cards/mahjong	0.7 (1.3)	0.6 (1.2)	< 0.001
Organized social activities	0.43 (1.0)	0.4 (0.9)	0.003
Paid job			0.02
Yes	6.6	5.6	
No	93.4	94.4	
Number of siblings frequently visited	0.9 (1.3)	0.5 (1.1)	< 0.001
Gender			0.97
Female	52.3	52.3	
Male	47.7	47.7	
Age			< 0.001
65–69	25.6	9.0	
70–74	21.9	10.0	
75–79	18.0	11.2	
80–84	11.3	9.6	
85–89	12.2	19.6	
90 +	11.1	40.6	
Residence			< 0.001
Rural	61.3	55.6	
Urban	38.7	44.4	
Education			< 0.001
Illiterate	52.1	58.2	
Literate	47.9	41.8	
Marital status			< 0.001
Unmarried	46.2	67.5	
Married	53.8	32.5	
Self-perceived economic conditions			0.13
Poor	14.9	16.0	
Fair	68.8	67.0	
Good	16.3	17.0	
Number of living children	3.9 (1.8)	3.5 (1.9)	< 0.001
Number of chronic diseases	1.2 (1.3)	1.2 (1.3)	0.68
Physical exercise			0.14
No	62.5	63.9	
Yes	37.5	36.1	
Cognitive ability index	20.6 (3.4)	18.8 (5.0)	< 0.001
Positive emotion index	11.6 (2.3)	11.3 (2.4)	< 0.001

^1^Significant difference in baseline characteristics between survivors in 2011 and those who dropped out between 2005 and 2011 was estimated using the Pearson χ^2^ test for categorical variables and the *T* test for continuous variables

## Abbreviations

ADL: Activities of daily living
CLHLS: Chinese Longitudinal Healthy Longevity Survey
MMSE: Mini-mental status examination
